# Photodynamic Therapy for Non-Melanoma Skin Cancers

**DOI:** 10.3390/cancers8100090

**Published:** 2016-10-04

**Authors:** Diana K. Cohen, Peter K. Lee

**Affiliations:** Department of Dermatology, University of Minnesota, Minneapolis, MN 55455, USA; leexx078@umn.edu

**Keywords:** photodynamic therapy, non-melanoma skin cancer, basal cell carcinoma, squamous cell carcinoma

## Abstract

Non-melanoma skin cancer (NMSC) is traditionally treated with surgical excision. Non-surgical methods such as cryotherapy and topical chemotherapeutics, amongst other treatments, are other options. Actinic keratosis (AKs) are considered precancerous lesions that eventually may progress to squamous cell carcinoma (SCC). Photodynamic therapy (PDT) offers an effective treatment for AKs, and is also effective for superficial basal cell carcinoma (BCC). Nodular BCC and Bowen’s disease (SCC in situ) have shown acceptable response rates with PDT, although recurrence rates are higher for these two NMSC subtypes. Methylaminolevulinate (MAL) PDT is a more effective treatment option than 5-aminolevulinic acid (ALA) PDT for nodular BCC. Several studies have shown that PDT results in superior cosmetic outcomes compared to surgical treatment. PDT is overall well-tolerated, with pain being the most common side effect.

## 1. Introduction

Non-melanoma skin cancer (NMSC) is the most common cancer of white-skinned individuals worldwide. The incidence is increasing and creating further problems for health care services [1]. Actinic keratoses (AKs) are lesions considered to be on a spectrum of clinical and histologic abnormalities that can ultimately progress to squamous cell carcinoma (SCC) [2]. The organ transplant population is at a particularly increased risk of NMSC, quantified as 65 to 250-fold for SCC, and as 10-fold for basal cell carcinoma (BCC) [3]. 

Several treatment modalities exist for AK, BCC, and SCC. The mainstay of treatment remains surgery via simple excision or Mohs Micrographic Surgery (MMS) for BCC and SCC. Photodynamic therapy (PDT) provides an alternative that has proven to achieve excellent cosmetic results for AK, BCC, and SCC. Methylaminolevulinate (MAL) and 5-aminolevulinic acid (ALA) are the two agents commonly utilized in clinical practice to perform PDT. Currently in the US, FDA approval for PDT is limited to treatment of AKs, whereas in the European Union (EU) and elsewhere worldwide, approval expands to the treatment of BCC and SCC in situ. The main aims of this review are to discuss the mechanism of PDT, and to address the clinical use of PDT for treatment of NMSC, including efficacy and tolerability.

## 2. Photodynamic Therapy

The medicinal basis of photodynamic therapy is centered around photooxidation occurring in a target tissue. Key components include the presence of a photosensitizer, oxygen, and light within the absorption spectrum of the photosensitizer [4]. Current clinical practice utilizes topical photosensitizers that are precursors of protoporphyrin IX (PpIX). After topical application of a photosensitizer to target tissue, there is an incubation period, followed by illumination with visible light to activate the photosensitizer. A type II photo-oxidation reaction produces reactive oxygen species (ROS), which destroy cell membranes and organelles, ultimately leading to cell death. The appropriate wavelength of light, concentration of sensitizer, and molecular oxygen level in the tissue are all critical for the efficacy of PDT [5].

### 2.1. Exploiting the Heme Synthesis Pathway 

Many years ago, a link was identified between a build-up of porphyrins, caused by defects in heme synthesis, and photosensitive conditions (porphyrias) [6]. Protophorphyrin IX (PpIX) was identified as the main culprit. Modern day PDT takes advantage of the photosensitive properties of PpIX by use of topical precursors that are taken up in tissue and converted to PpIX [7].

ALA formation occurs early on in the heme synthesis pathway and is the rate-limiting step in the pathway. Administration of exogenous ALA therefore bypasses the rate-limiting step [8]. ALA administered topically or systemically is non-selectively taken up into cells and metabolized into photosensitive PpIX by the resident enzyme machinery [8].

Although the uptake of ALA is non-selective, accumulation of PpIX in tumor cells may occur selectively because of alterations in enzymatic activity in the heme synthesis pathway. In neoplastic tissue, it is theorized that activity of porphobilinogen deaminase (PBGD) increases. PBGD synthesizes a precursor of PpIX and thus increases production of PpIX. The accumulation of PpIX in neoplastic tissue is further supplemented by decreased activity of ferrochelatase (FC), which converts PpIX to heme [9,10]. Another postulated mechanism of selectivity for topical ALA relates to the altered stratum corneum of tumoral skin. ALA has been found to penetrate more rapidly into superficial BCC tissue compared with adjacent surrounding normal skin as measured by microdialysis catheters [11].

### 2.2. Topical Photosensitizing Drugs (ALA and MAL)

ALA and MAL are the most commonly used agents with PDT in clinical practice. Although these two molecules bear a similar mechanism of action as prodrugs that lead to production of photoactive PpIX, there are notable differences.

ALA is a hydrophilic molecule and is used to treat more superficial lesions due to its modest tissue penetration [12]. Uptake of ALA into tissue occurs via BETA transporters (GABA transporters) in an active manner [13]. ALA is approved for use with blue light PDT as Levulan^®^ Kerastick^®^ (DUSA Pharmaceuticals Inc., Wilmington, MA, USA), administered in a 20% solution. Approval has been granted for treatment of AKs in the US, Korea, Mexico, Brazil, Argentina, Chile, and Columbia. The FDA-approved protocol for Levulan^®^ PDT involves a 14 to 18 hour incubation after application of the product. Incubation is then followed by illumination with blue light (400–450 nm, 10 J/cm^2^) with BLU-U^®^ (Blue light PDT illuminator, DUSA Pharmaceuticals Inc, Wilmington, MA, USA) [14]. ALA is also approved for use with red light as Ameluz^®^ (Biofrontera AG, Wakefield, MA, USA), administered in a 10% gel, for treatment of AKs in the U.S. and the Europe Union (EU). Ameluz^®^ is applied and then incubated for 3 hours under an occlusive dressing, then illuminated with the BF-RhodoLED^®^ PDT lamp (635 nm, 37 J/cm^2^) (Biofrontera AG, Wakefield, MA, USA).

In clinical practice, ALA is often used off-label because of decreased incubation times. A study of this practice showed that ALA/PDT at incubation times of 1, 2, and 3 hours was superior compared to vehicle/PDT. Complete clearance rate of AKs for ALA/PDT ranged from 17% to 30% at week 12, compared with 2% of the vehicle/PDT group. Median AK clearance rates for the ALA/PDT group were 68% to 79%, compared with 7% of the vehicle/PDT group [15].

MAL, a methyl ester of ALA, was developed to improve upon the tissue penetration achieved by ALA. The ester molecule is lipophilic, which results in greater penetration and, in turn, a higher intracellular accumulation of PpIX [12]. In contrast to the active transport of ALA, MAL uptake is thought to occur by passive diffusion and non-polar amino acid transport [16]. MAL is approved as Metvix™ (Galderma Laboratories, LP, Fort Worth, TX, USA), a 16.8% cream, for the treatment of AKs in the U.S., although it is not currently available on the U.S. market. Metvix™ is approved in the EU for the treatment of AKs, superficial and nodular BCC, and Bowen’s disease [17]. Approved administration of MAL cream involves application, occlusion for 3 hours, saline wash, then illumination with Aktilite^®^ CL 128 LED narrow-band red light (630 nm, 37 J/cm^2^) (Galderma Laboratories, LP, Fort Worth, TX, USA).

MAL also has shown a preference for neoplastic cells. Although there are no selectivity studies comparing MAL and ALA directly, it is presumed that MAL shows a greater selectivity than ALA, likely attributed to the differing uptake mechanisms [18]. A 10-fold difference was observed between lesional skin and normal skin in the first hours after application of MAL. Selectivity may be the reason behind findings that use of MAL/PDT compared to ALA/PDT is associated with decreased pain [19,20]. Kasche et al. found that 54% of patients discontinued illumination prior to finishing ALA/PDT, compared to 14% in the MAL/PDT group [20]. Another study found mean pain scores to be higher with ALA/PDT compared to MAL/PDT during and immediately following illumination, although no significant difference was noted at 24 hours after illumination. Non-selective GABA transport of ALA into nerve cells is also postulated to play a role in the increased pain with ALA/PDT [19].

### 2.3. Light Source

Numerous light sources have been used in conjunction with PDT over the years. Alexiades-Armenakas summarized the characteristics of the ideal light source for PDT to treat NMSC, citing 5 key points: the light source should “(1) be absorbed well by the photosensitizer, (2) achieve a desirable penetration depth, thereby reaching its target, (3) have an adequate fluence and duration to drive the PDT reaction, (4) be rapid to administer, and (5) have minimal discomfort, be purpura-free, with minimal erythema, rapid recovery, and no risk for crusting or dyspigmentation [21]”.

LED light sources in the red and blue range are commonly used in the US and Europe for cutaneous PDT, and are government approved devices [14,17]. Laser light has also been explored for use with PDT [21,22,23,24,25,26]. Recently, Kessels et al. reported a prospective split-face study comparing LED–PDT to Pulsed Dye Laser (PDL)-PDT with long-term follow-up of 12 months for treatment of AKs [26]. Results showed no significant difference in the mean change in number of AKs, indicating PDL-PDT as an effective treatment for AK [26]. Advantages of PDL include decreased pain and discomfort during the procedure, and faster treatment times [21,22,26]. However, there are disadvantages to PDL–PDT, such as high cost, availability of the device, and need for expertise to use the device [26].

It is often assumed that a coherent light source would have a greater depth of penetration as compared to a noncoherent light source, and thereby the coherent source would achieve better clearance of thick skin tumors. However, studies have not supported this assumption. Coherence appears to be lost within <1 mm of penetration into the skin [27]. In addition, noncoherent light has shown moderate penetration depths of up to 5 mm for 630 nm light, and up to 1–2 cm for 700–800 nm light [28,29,30]. Results from studies using PDL–PDT on superficial and nodular BCC and Bowen’s disease have been mixed [22,31,32]. Coupled with the disadvantages of PDL-PDT as stated above, it is no surprise that most practitioners opt to perform LED–PDT given its convenience and similar efficacy.

### 2.4. Tolerability and Side Effects

Pain is a major and serious adverse event during ALA/PDT and MAL/PDT. Pain can lead to incomplete treatments and foregoing of repeat treatments [33]. As above, MAL/PDT is associated with decreased pain levels compared to ALA/PDT [19,20]. Multiple interventions have been studied to attempt to reduce pain during PDT. Morphine 0.3% gel, applied 15 minutes prior to illumination, did not result in a significant reduction in pain as compared to a placebo gel [34]. Cooling and pauses during PDT, in combination, were effective in achieving a considerable reduction in pain [35]. A mixture of nitrous oxide/oxygen inhaled during PDT led to an overall reduction in pain of 55.2%, and an 82% decrease in therapy interruptions [33]. Studies have demonstrated that PDT using PDL coherent light, as compared to LED non-coherent light, results in less pain and an increase in willingness to undergo subsequent PDT treatments [26].

Burning and prickling sensations are also common side effects of PDT. These sensations are usually mild to moderate in intensity and transient [36]. A phase IV clinical trial of ALA/PDT used to treat AKs found 96% of patients experienced stinging/burning at 6 and 11 minutes into the light treatment. 10% reported the intensity as severe. Erythema and edema immediately after light treatment were commonly noted, but resolved to levels less than baseline at the 1 month follow-up. Hypo- and hyper-pigmentation are potential adverse effects of treatment that were each noted in 5% of lesions or less. More often, pigmentary changes noted prior to treatment resolved at the last follow-up post-treatment [37].

Allergic contact dermatitis is an extremely rare but notable adverse effect. A study by Cordey and Ibbotson found that 10 patients out of 1532 patients treated from 1998 to 2015 had positive patch test reactions to PDT prodrugs, for a rate of 0.65% [38]. Testing of the base preparations without active ingredients was not performed, however.

## 3. Actinic Keratoses

PDT is a frequently utilized treatment for AKs and, in some instances, considered the first-line treatment. Several studies of efficacy have been conducted for both ALA/PDT and MAL/PDT in treatment of AKs. A phase III clinical trial of ALA/PDT for the treatment of multiple AKs of the face and scalp found 89% of patients had 75% or more of their AKs treated by week 12 [39]. In a phase IV clinical trial to assess longer term results, ALA/PDT resulted in an overall lesional recurrence rate of 24% judged by clinical exam. Of the 162 lesions clinically diagnosed as recurrent AKs, 139 lesions were biopsied. The other lesions were either lost to follow-up (16) or cleared (7). 91% of biopsied lesions were confirmed histologically as AK, 7% were found to be SCC, and 0.7% BCC. Recurrent and non-responding lesions did not show an anatomic predilection, as they were found widely distributed on the face and scalp [37]. These studies were conducted with the FDA-approved incubation regimen of illumination occurring with blue light for 14–18 hours after ALA application. Studies have suggested efficacy rates approaching those in the larger clinical trials for shortened incubation times, as short as 1 hour, which is off-label use [15,37]. The newer gel formulation of ALA showed patient complete clearance rates of 78.2%, significantly higher than MAL cream, at the 3 month follow-up point.

MAL/PDT has also demonstrated success in treatment of AKs. Use with red light showed an 89% complete lesion response rate compared to a 38% placebo rate at 3 months. Excellent or good cosmetic results were seen in over 90% of patients treated with MAL/PDT [40]. Tarstedt et al. analyzed MAL/PDT for treatment of thin versus thick AKs and found that a single treatment was effective for thin AKs, whereas a repeat treatment 1 week after the initial treatment was more effective for thick lesions [41].

## 4. Basal Cell Carcinoma

A number of studies have assessed the efficacy, cosmetic results, and recurrence rates of BCC treated with PDT [42,43,44,45,46,47,48,49,50,51,52,53]. MAL/PDT is approved in the EU for treatment of BCC, but remains off-label in the United States. PDT has shown to generally be more effective for superficial BCC as compared to nodular BCC, and also for smaller lesions <2 cm [48,49,50,52]. However, when considering use of PDT for treatment of larger and nodular BCC, MAL/PDT specifically has shown more promise as compared to ALA/PDT. Whereas ALA/PDT was found to have a 30.7% recurrence rate for treatment of nodular BCC in one study [52], MAL/PDT showed a 14% recurrence rate for treatment of nodular BCC in another study [51]. Christensen et al. conducted the longest follow-up of any study to date, which spanned 10 years. The overall complete response rate was 75% for all subtypes of BCC treated with ALA/PDT, with a 60% complete response after one treatment and 87% response after two treatments [45]. Further long term studies are warranted to better assess the effectiveness of PDT on BCC.

Compared to surgical excision, PDT appears to result in higher BCC recurrence rates [42,47,51,52,54]. Rhodes et al. found the recurrence rate for primary nodular BCC to be 14% with PDT and 4% with surgical excision at the 5-year follow-up point [51]. A meta-analysis of PDT versus surgical excision by Zou et al. concluded that PDT is comparably effective to excision for treatment of BCC, but with increased risk of recurrence [54]. Multiple studies have noted that PDT compared to excision results in better cosmetic outcomes [42,47,51].

Vinciullo et al. specifically examined MAL/PDT for “difficult-to-treat” BCC, defined as large lesions, lesions in the H-zone, or lesions in patients with a high risk of surgical complications [43]. Failure rate of treatment was 18% at 12 months and 24% at 24 months, with a cosmetic outcome of excellent or good in 84% of patients at 24 months. The authors concluded that MAL/PDT is an attractive treatment option for the subset of “difficult-to-treat” BCCs given that surgical treatment would have been extensive and resulted in a worse cosmetic outcome.

A couple of recent small studies comparing red light LED-PDT with PDL-PDT for treatment of BCC have demonstrated slightly better clearance and recurrence rates with red light LED-PDT. A small pilot study with 6 patients, each with 1 large superficial BCC (average diameter of 3.5 cm), was conducted using a split lesion design. One half of each lesion was treated with 630 nm LED-PDT, and the other half was treated with 595 nm PDL-PDT, both using MAL as the photosensitizer. 5 patients achieved complete response with 630 nm LED–PDT, but an incomplete response with PDL-PDT. 1 patient did not respond to either treatment [32]. Another study with 15 patients, using an intra-individual split design, with 630 nm LED-PDT and 585 nm PDL-PDT on similarly sized BCCs (nodular or superficial), showed similar clearance rates with both treatments, but higher recurrence rates with PDL-PDT [31].

For small and superficial BCC, PDT is a reasonable option for treatment, although it is not considered first line. Larger and nodular BCC may also be treated with MAL/PDT, however the risk of recurrence must be weighed against the gains in cosmetic outcomes when compared to surgical excision.

## 5. Squamous Cell Carcinoma of the Skin

Use of MAL/PDT for treatment of Bowen’s disease, or squamous cell carcinoma in situ, is approved in several European countries. The dosing regimen for MAL/PDT in Europe consists of two treatments 7 days apart, repeated at 3 months, as needed [55].

Historically, there has been controversy regarding use of PDT for Bowen’s disease, given recurrence rates and the potential for squamous cell carcinoma to metastasize. Fink-Puches et al. [56] studied ALA/PDT for superficial SCC, defined as SCC confined to the papillary dermis, and projected a disease-free rate of just 8% at 36 months after treatment. These lesions were not in situ, and subsequent studies have shown better response rates. A retrospective study of 31 Bowen’s lesions treated with MAL/PDT in Brazil found 14 of 31 lesions recurrent, for a rate of 53.8%, with a mean follow-up of 43.5 months [57]. Comparing MAL/PDT to cryotherapy and 5-fluorouracil showed similar response rates with all treatments used against SCC in situ at the 12 month follow-up point [58]. A study comparing ALA/PDT to MAL/PDT with 9 and 18 Bowen’s disease lesions, respectively, showed an 89% and 78% response rate, respectively, at approximately 6 months after treatment [59].

One study using 585 nm PDL-PDT for treatment of Bowen’s disease, with ALA as the photosensitizer, demonstrated a complete clinical response rate of 82% at 1-year follow-up, which is in-line with LED-PDT response rates in other studies. Morbidity after the procedure, however, was high, as 1 patient (out of the 13 patients in the study) developed cellulitis at the site of treatment, 8 patients had prolonged crusting lasting 8 weeks, and 4 patients had prolonged discomfort lasting 6 weeks after treatment.

PDT shows promise for treatment of Bowen’s disease, but larger studies with longer follow-up are needed to better assess response rates. Caution should be used with this treatment, given the potential for squamous cell carcinoma to metastasize. Similar to treatment outcomes of BCC, cosmetic outcomes have overall been good in most patients [58].

## 6. Organ Transplant Recipients

Organ transplant recipients on long-term immunosuppressive therapy are at an increased risk of NMSC, particularly SCC. PDT has proven useful in reducing the incidence of AKs and SCCs in this special population. Willey et al. carried out cyclic ALA/PDT, defined as treatments at 4 to 8 week intervals over a 2-year period, on twelve patients who were solid organ transplant recipients. There was a 95% mean reduction in SCC lesion count at 24 months post-treatment, compared to 1 month pre-treatment [60]. Wennberg et al. found that repeat MAL/PDT treatments 1 week apart at months 0, 3, 9, and 15 reduced the occurrence of new AKs in this special population [61].

## 7. Conclusions

Given the increasing incidence of NMSC, therapies are continually sought after to optimize patient comfort and cosmetic outcomes while still achieving acceptable response rates. PDT offers an attractive alternative to surgical treatment of NMSC, as well as an alternative to non-surgical treatments such as cryotherapy and 5-fluorouracil. PDT is well-tolerated, with pain during and shortly after treatment being the main adverse effect. MAL appears be associated with lower pain levels than ALA, which may be due to its greater selectivity for neoplastic lesions. New strategies, such as cooling and inhalation of a nitrous oxygen/oxygen mixture, are promising treatments to minimize pain. MAL also requires shorter incubation times compared to ALA, according to the FDA-approved treatment regimen. PDL–PDT is another option for patients who cannot tolerate LED–PDT due to pain, albeit PDL–PDT is less widely available, higher in cost, and not approved for treatment of NMSC.

PDT utilizing ALA and MAL is a proven and even first line treatment for AK. PDT has also demonstrated efficacy in treatment of BCC and SCC in situ, although recurrence rates higher than those of standard surgical treatments preclude first-line use of PDT for these indications. Studies with MAL/PDT for superficial BCC offer acceptable response rates to consider it a reasonable therapeutic option for patients who are not surgical candidates or who do not desire surgery. PDT should be utilized with caution for nodular BCC and Bowen’s disease given the risk of recurrence.
